# Construction and analysis of a novel ferroptosis‐related gene signature predicting prognosis in lung adenocarcinoma

**DOI:** 10.1002/2211-5463.13288

**Published:** 2021-10-16

**Authors:** Jing Zhou, Xinyue Wang, Zhaona Li, Richeng Jiang

**Affiliations:** ^1^ Tianjin Medical University Cancer Institute & Hospital National Clinical Research Center for Cancer Tianjin China; ^2^ Key Laboratory of Cancer Prevention and Therapy Tianjin China; ^3^ Tianjin’s Clinical Research Center for Cancer China; ^4^ Department of Thoracic Oncology Tianjin Lung Cancer Center Tianjin Cancer Institute & Hospital Tianjin Medical University China

**Keywords:** ferroptosis, lung adenocarcinoma, overall survival, prognostic risk signature

## Abstract

Ferroptosis is a newly discovered, iron‐dependent, nonapoptotic form of programmed cell death that plays an important role in the development of lung adenocarcinoma (LUAD). In this study, ferroptosis‐related genes (FRGs) were identified from the FerrDb dataset, and the mRNA expression profiles and corresponding clinical data of LUAD patients were downloaded from the University of California, Santa Cruz (UCSC) databases. Data from LUAD patients from the Gene Expression Omnibus (GEO) dataset were used as the verification set. Cox and Lasso regression analyses were used to screen the FRGs with prognostic value, and six prognostic‐related FRGs were selected to construct prognostic risk score signatures. Immunohistochemistry was utilized to manifest the differential expression of six FRGs in tumor and normal tissues at the protein level. Functional enrichment analysis indicated that FRGs were mainly enriched in ferroptosis‐related pathways. Patients were divided into high‐ and low‐risk groups based on the median risk score. The Kaplan–Meier survival curves confirmed that patients with a high score had significantly worse overall survival. Receiver operating characteristic (ROC) curves proved that the prognostic signature has good sensitivity and specificity for predicting the prognosis of LUAD patients. Nomogram analysis showed that the prognostic signature has potential independent prognostic value. Moreover, the prognostic signature has been shown to be significantly associated with some clinical features (T stage, N stage, tumor stage, and survival status) as well as many immune‐activity‐related genes and immune‐checkpoint‐related genes. In conclusion, we constructed a prognostic signature consisting of six FGRs, which can provide a reference for predicting the prognosis of LUAD patients.

## Graphical abstract

A bioinformatic approach was used to screen ferroptosis‐related genes (FRG) with prognostic value, and a prognostic signature consisting of 6 FRGs was constructed in LUAD. Performance evaluation indicated that the predictive performance of the prognostic signature was stable and had independent prognostic value, which is expected to provide reference for individualized diagnosis and treatment of LUAD patients.

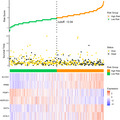

**Abbreviations**
AUC: area under the curve
DEGs: differentially expressed genes
FRGs: ferroptosis‐related genes
GEO: The Gene Expression Omnibus
GO: Gene Ontology
IHC: immunohistochemistry
KEGG: Kyoto Encyclopedia of Genes and Genomes
LASSO: least absolute shrinkage and selection operator
LUAD: lung adenocarcinoma
NSCLC: non‐small‐cell lung cancer
OS: overall survival
ROC: receiver operating characteristic
ROS: reactive oxygen species
TCGA: The Cancer Genome Atlas

Lung cancer is the leading cause of cancer‐associated mortality across the world, with a 5‐year survival rate of about 15%. Non‐small‐cell lung cancer (NSCLC) accounts for 75%–80% of all lung cancer [1]. Among them, lung adenocarcinoma (LUAD) is the most common NSCLC subtype among nonsmokers and women and has several known risk factors, including second‐hand smoking, pollution and occupational carcinogens, and with inherited genetic susceptibility [2]. Although great advances have been made in chemotherapy and targeted therapies for lung cancer, overall survival (OS) is still low for most patients [3]. One of the main reasons for this is that most patients are diagnosed at an advanced stage. At present, the commonly used indicators to predict the prognosis of LUAD include tumor size, metastasis, and tumor mutational burden [4]. However, tumor tissues are highly heterogeneous, and even patients with the same TNM stage still have a great difference in treatment effect and prognosis. Sometimes, relying solely on the above indicators cannot accurately predict the prognosis of patients with poor specificity. Therefore, we need to explore new biomarkers, which can assist the commonly used predictive indicators, and reliably evaluate the prognosis and survival of tumor patients, so as to provide a basis for the individualized diagnosis and treatment of LUAD.

Ferroptosis is a newly discovered, iron‐dependent, nonapoptotic form of programmed cell death characterized by intracellular accumulation of reactive oxygen species (ROS) [5, 6]. The essence of ferroptosis is the metabolic disorder of intracellular ROS [7]. Under the catalysis of iron ions, metabolic abnormalities occur, the antioxidant capacity of cells is weakened, and the accumulation of ROS leads to oxidative death of cells [8].

Over the past few years, with the in‐depth development of research, emerging evidence shows that ferroptosis is related to the occurrence and development of a variety of diseases, including Parkinson's disease, head and neck carcinoma, breast cancer, and blood diseases 9, 10, 11, 12. Recent studies indicate that inducing ferroptosis is a promising therapy for the treatment of cancer, especially for malignancies which are resistant to traditional therapies [13]. Meanwhile, many ferroptosis‐related genes (FRGs) such as GPX4, NOX1, ACSL4, and PTGS2 have also been discovered successively [14, 15, 16, 17. Yang *et al*. [18] found that downregulated GPX4 expression makes cells more sensitive to ferroptosis, whereas upregulated GPX4 expression induces tolerance to ferroptosis. The ferroptosis‐related gene NFS1 has been detected highly expressed in LUAD cells [19]. There is also considerable interest in ferroptosis involvement and application in LUAD. Dysregulation of iron is gradually being recognized as a risk factor for lung cancer. According to research, ferritin levels of patients with NSCLC are significantly elevated [20, 21]. In NSCLC, erastin was recently found to stimulate the expression of p21 and Bax by enhancing and activating p53, inhibit SLC7A11 activity, subsequently stimulate ROS accumulation, and induce ferroptosis as well as apoptosis in A549 cells [22]. LUAD ferroptosis is promoted by NFS1 suppression, when ROS accumulation is present within the cell, and has little effect, when NFS1 is inhibited on its own without excessive ROS [19]. These studies suggest that ferroptosis does exist in LUAD and that targeting ferroptosis may break through some of the limitations of traditional anticancer therapies. Hence, a comprehensive study of ferroptosis would provide a novel treatment therapy for patients with LUAD, including those with drug‐resistant cancer.

In recent years, prognostic signature based on multiple genes has been widely studied and used to predict the prognosis and treatment of various tumors, such as lung cancer, ovarian cancer, and hepatocellular carcinoma [23, 24, 25. The predict performance of multiple gene models is even better than that of histopathological diagnosis in some cancer types. Despite this, no study has been performed to determine whether a ferroptosis‐related gene prognostic signature could predict the outcome of LUAD. The purpose of this study is to fill this blank and widen the potential of diagnosis and therapy of LUAD. In this work, FRGs were collected, and bioinformatics was used to screen FRGs with prognostic value through univariate Cox regression analysis and Lasso regression, and a LUAD prognostic signature consisting of 6 FRGs was constructed. Performance evaluation and validation of external datasets indicated that the predictive performance of the prognostic signature was stable and had independent prognostic value, which is expected to provide reference for individualized diagnosis and treatment of LUAD patients.

## Materials and methods

### Collection of datasets and FRGs

The levels 3 mRNA expression profile (HTSeq‐FPKM) and corresponding clinical information (including age, gender, TNM stage, and OS) of 585 LUAD sample were collected from the UCSC Xena (https://xenabrowser.net/datapages/). A total of 497 The Cancer Genome Atlas (TCGA)‐LUAD samples were included after excluding samples with incomplete clinical information. The GSE31210 and GSE72094 gene expression profiles and the corresponding clinical information (including age, gender, stage, and OS) were downloaded from The Gene Expression Omnibus (GEO; https://www.ncbi.nlm.nih.gov/geo/) database. In this study, TCGA‐LUAD was regarded as a training set, the GSE31210 and GSE72094 were treated as independent validation sets, respectively. Meanwhile, 259 FRGs were collected from the FerrDb database (http://www.zhounan.org/ferrdb), which is the world’s first database to collection ferroptosis regulators and markers and ferroptosis‐disease associations [26]. All research datasets in this study are from public databases, and no ethical approval is required.

### Construction and validation of prognostic signature

Utilizing univariate Cox regression analysis to identify FRGs with prognostic value, the gene with *P* < 0.05 was considered statistically significant and could be included in least absolute shrinkage and selection operator (LASSO) regression [27]. LASSO regression can better solve the influence of multicollinearity in regression analysis. The least absolute shrinkage and LASSO regression algorithm for gene selection, using 10‐fold cross‐validation, the above analysis uses the r software package ‘glmnet’. The prognostic risk signature is constructed by using the corresponding genes when penalty parameter (λ) is taken as the minimum value, and the model can reach the optimal. The risk score was calculated for each patient according to the following formula:

$$\text{risk}\;\text{score}\;=\;\sum_{i=1}^{n}\left[(\text{expGene})i\;\times \;\text{coef}i\right]$$

(expGene is the gene expression value, coef is the regression coefficient, and n represents the total number of genes). Risk score of each TCGA‐LUAD patient was calculated according to the above formula, and then, patients were divided into high‐risk and low‐risk groups based on the median value of risk score. The GSE31210 and GSE72094 serve as external validation sets to validate the predictive performance of the risk signature. The Kaplan–Meier survival analysis with log‐rank test was plotted to compare the OS difference between above two groups. *P* < 0.05 was considered as statistically significant. The risk score distribution, survival status distribution, and time‐independent receiver operating characteristic (timeROC) curve were performed to compare the predictive accuracy of each gene and risk score. The higher area under the curve (AUC), the better the signature's performance.

### Development and validation of a nomogram

Univariate and multivariate Cox regression analyses were performed for risk signature and clinical characteristics, such as age, sex, and stage, to determine whether the signature had independent prognostic value. If risk signature was significantly different in univariate and multivariate COX analyses, it suggested that risk score might be an independent prognostic factor. A nomogram was developed based on the results of multivariate Cox regression analysis to predict the 1‐, 3‐, and 5‐year OS in patients with LUAD. The nomogram provided a visualization of these risk factors, which can be applied to speculate the OS for an individual patient by calculating a risk factor score through “rms” r package.

### Prognostic and protein level expression of FRGs

To investigate the prognosis and potential therapeutic value of six FRGs in LUAD patients, the prognosis of their different expression was analyzed by KM survival curve, and the different expression of six genes in tumor and normal tissues was verified by immunohistochemistry (IHC) in the Human Protein Atlas (https://www.proteinatlas.org) [28].

### Functional enrichment analysis

To explore the potential molecular mechanism of the prognostic signature, the “limma” r package was used to screen differentially expressed FRGs of high‐risk and low‐risk score groups, with a cutoff value set at *P* < 0.05 and | Log2FC| > 0.5. The “clusterProfiler” R package was utilized for Gene Ontology (GO) and Kyoto Encyclopedia of Genes and Genomes (KEGG) pathway enrichment analysis of differentially expressed FRGs, and the *P* < 0.05 was considered as statistically significant terms [29, 30].

### Validate the immunocorrelation and clinical characteristic correlation of prognostic signature

In order to further verify the immune correlation and clinical characteristic correlation of risk score, we first selected PRF1, TNF, GZMA, TBX2, CXCL9, CXCL10, GZMB, CD8A, and IFNG as immune‐activity‐related signatures and PDCD1, HAVCR2, CD274, LAG3, CTLA4, and IDO1 as immune‐checkpoint‐relevant signatures, applying the Spearman coefficient and Wilcoxon rank‐sum to comprehensively analyze the relationship between risk score and immune‐checkpoint‐relevant signatures and immune‐activity‐related signatures. The risk score was then analyzed to determine whether it also predicted prognosis for patients with different clinical characteristics, including sex, age, tumor size, presence of lymph node or distant metastasis, tumor stage, and survival status.

## Results

### Construction of prognostic signature

**Fig. 1:**
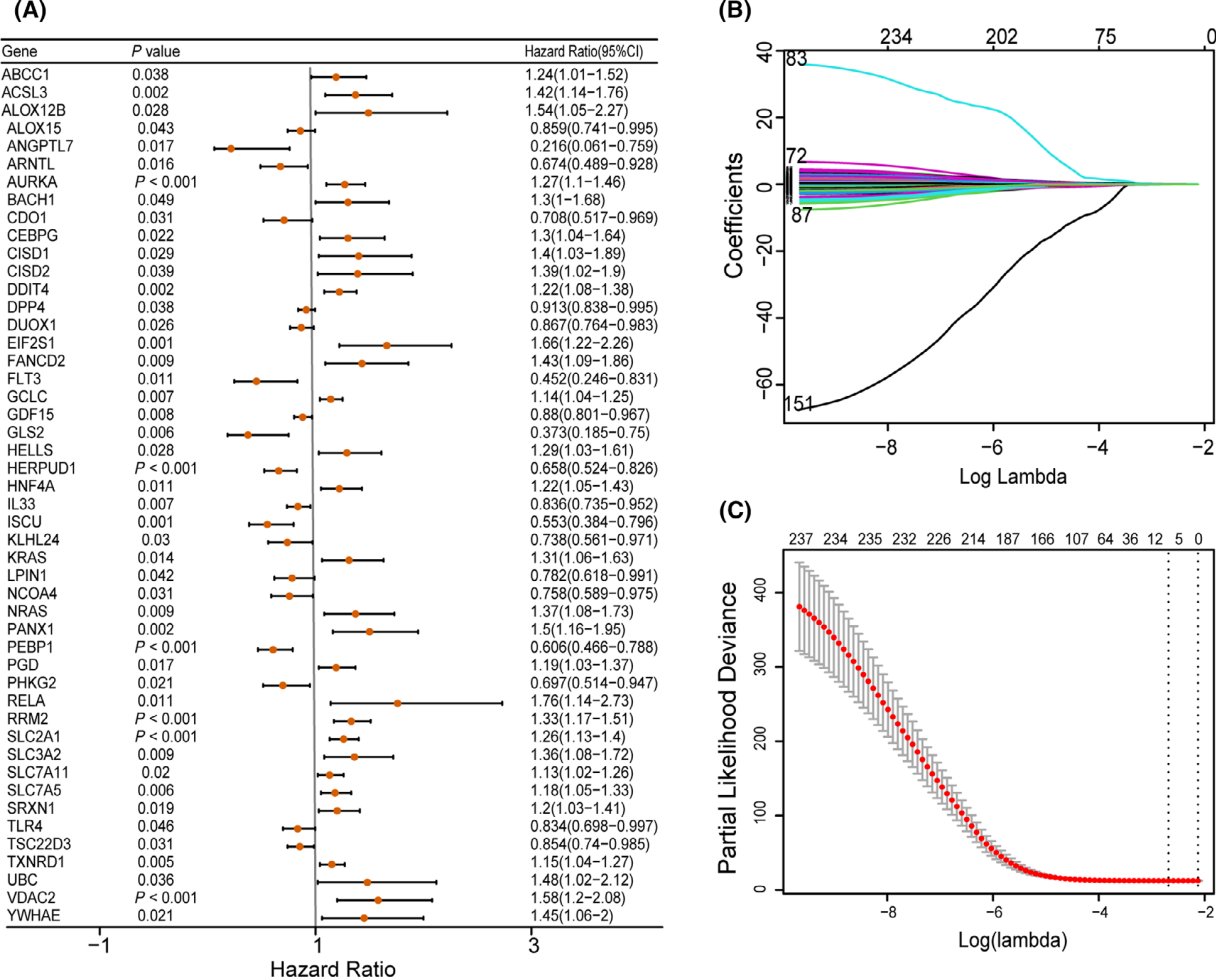
Construction of prognostic signature. (A) The forest plots of univariate Cox regression analysis. (B) LASSO coefficient profiles of the 6 FRGs. (C) Select the optimal value of λ by LASSO regression. Univariate Cox regression analysis using the ‘survival’ R4.0.3 package. Error bars indicate confidence interval (CI).

**Fig. 2:**
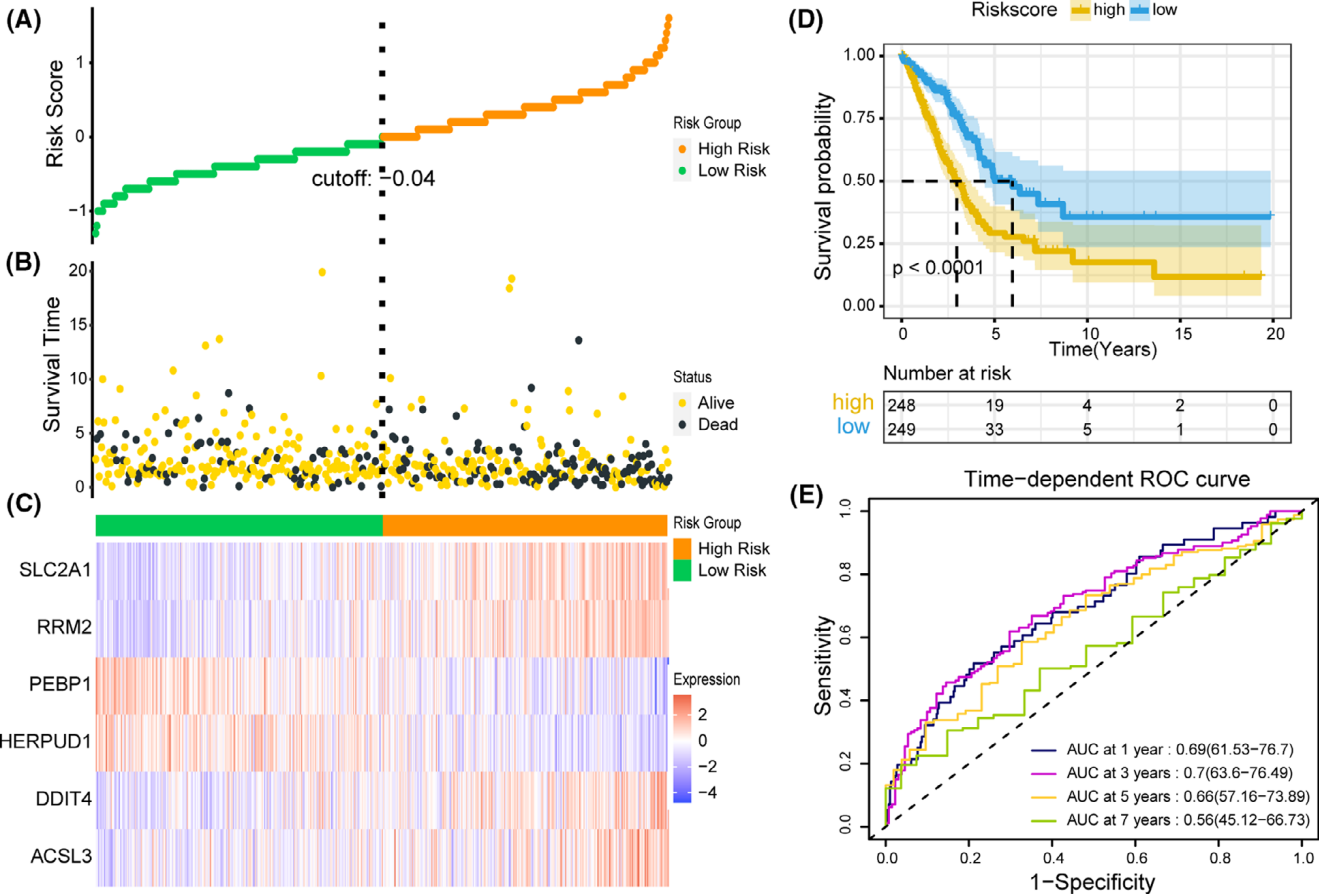
Performance evaluation of prognostic signature in training set. (A) The curve of risk score distribution in training set. (B) The curve of survival status distribution in training set. (C) Heatmap of the expression profiles of prognostic signature genes in high‐risk score and low‐risk score group in training set. (D) Kaplan–Meier survival analysis of the prognostic signature in training set with two‐sided log‐rank test. (E) Time‐dependent ROC analysis of the prognostic signature for predicting the 1‐, 3‐, 5‐, and 7‐year OS in training set.

A total of 239 FRGs were extracted from the TCGA‐LUAD dataset, and univariate Cox regression analysis showed that 48 FRGs were significantly associated with OS in LUAD patients (*P* < 0.05, Fig. 1A). LASSO regression analysis was performed on 48 FRGs to identify more stable genes. As shown in Fig. 1B,C, the dotted line marked the minimum Log (λ) value, the coefficients were not zero, and corresponding six gene was the best signature gene. According to the result of LASSO regression, ACSL3 (Acyl‐CoA synthetase long chain family member 3), DDIT4 (DNA damage inducible transcript 4), HERPUD1 (homocysteine‐inducible ER protein with ubiquitin‐like domain 1), PEBP1 (phosphatidylethanolamine binding protein 1), RRM2 (ribonucleotide reductase regulatory subunit M2), and SLC2A1 (solute carrier family 2 member 1) were selected to build prognostic signature of LUAD. The upregulated ACSL3, DDIT4, RRM2, and SLC2A1 with HR > 1 were considered as oncogenes, whereas the downregulated HERPUD1 and PEBP1 with HR < 1 were regarded as tumor suppressors. The risk score = (0.3333 × expression of ACSL3) + (0.1616 × expression of DDIT4) + (−0.3176 × expression of HERPUD1) + (−0.2531 × expression of PEBP1) + (0.1387 × expression of RRM2) + (0.0145 × expression of SLC2A1). Based on the expression levels of 6 FRGs and regression coefficients, the risk score of each TCGA‐LUAD patient was calculated, and patients were divided into high‐risk group (*n* = 248) and low‐risk group (*n* = 249) according to the median risk score. We further studied the performance of prognostic signature in predicting the prognosis of TCGA‐LUAD patients, and the visualization results were shown in figure. Orange represents the high‐risk score group, and green represents the low‐risk score group (Fig. 2A). LUAD patients in the high‐risk score group had a higher mortality rate than those in the low‐risk score group, suggesting that the high‐risk score group was more likely to have a poor prognosis (Fig. 2B). ACSL3, DDIT4, RRM2, and SLC2A1 were highly expressed in the high‐risk score group, suggesting that high expression was positively correlated with high risk, while the expression of PEBP1 and HERPUD1 was low in the high‐risk score group, indicating a positive correlation between low expression and high risk (Fig. 2C). Kaplan–Meier survival curves showed that patients with high‐risk score group had a lower OS than patients with low‐risk score group (*P* < 0.0001; Fig. 2D). The results of timeROC curve manifested that the AUC of 1 year is 0.69, 3 years is 0.70, 5 years is 0.66, and 7 years is 0.56 (Fig. 2E). The above evaluation results indicated that the predictive performance of the LUAD prognostic signature was not satisfactory (AUC_max_ = 0.70), and further verification was needed by validation set.

### Validation of prognostic signature in GEO datasets

**Fig. 3:**
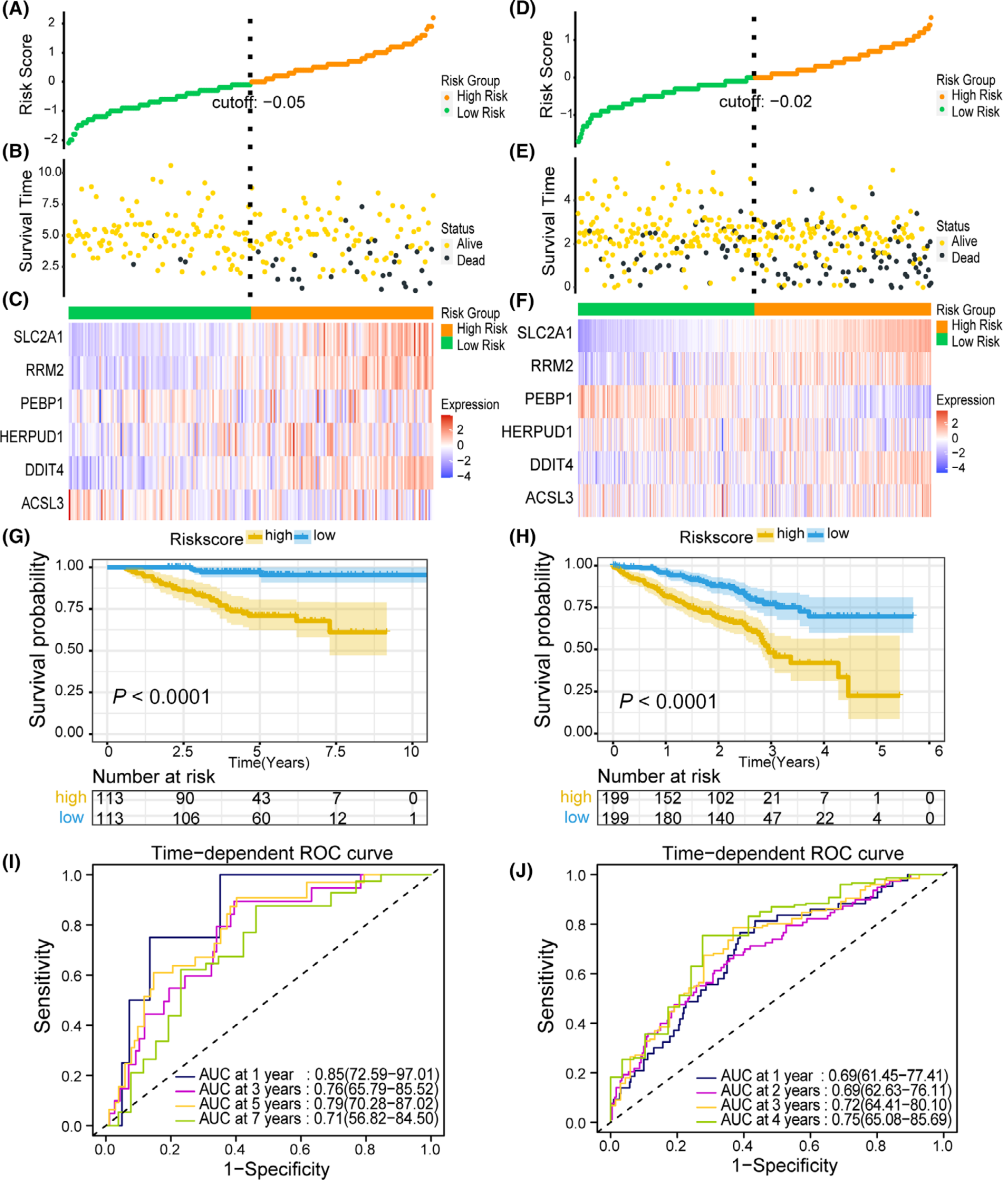
Performance evaluation of prognostic signature in validation set. (A) The curve of risk score distribution in GSE31210. (B) The curve of survival status distribution in GSE31210. (C) Heatmap of the expression profiles of prognostic signature genes in high‐risk score and low‐risk score groups in GSE31210. (D) The curve of risk score distribution in GSE72094. (E) The curve of survival status distribution in GSE72094. (F) Heatmap of the expression profiles of prognostic signature genes in high‐risk score and low‐risk score groups in GSE72094. (G) Kaplan–Meier survival analysis of the prognostic signature in GSE31210 with two‐sided log‐rank test. (H) Kaplan–Meier survival analysis of the prognostic signature in GSE72094 with two‐sided log‐rank test. (I) Time‐dependent ROC analysis of the prognostic signature for predicting the 1‐, 3‐, 5‐, and 7‐year OS in GSE31210. (J) Time‐dependent ROC analysis of the prognostic signature for predicting the 1‐, 2‐, 3‐, and 4‐year OS in GSE72094.

GSE31210 and GSE72094 datasets were selected from the GEO database and used as external validation sets. The distribution of risk score, survival status, and 6 gene expression profiles of the above two validation sets were almost consistent with the previous studies, and more patients died in the high‐risk score group than in the low‐risk score group (Fig. 3A–F). The Kaplan–Meier survival curves for both test and validation sets confirmed that patients with high score had significantly worse OS than patients with low score (*P* < 0.0001; Fig. 3G, *P* < 0.0001; Fig. 3H). The AUC of GSE31210 was 1 year is 0.85, 3 years is 0.76, 5 years is 0.79, and 7 years is 0.71 (Fig 3I). The AUC of GSE72094 was 1 year is 0.69, 2 years is 0.69, 3 years is 0.72, and 4 years is 0.75 (Fig. 3J). The evaluation results of the two validation sets suggested that the signature has good sensitivity and specificity for predicting the prognosis of LUAD.

### Independent prognostic value of prognostic signature

**Fig. 4:**
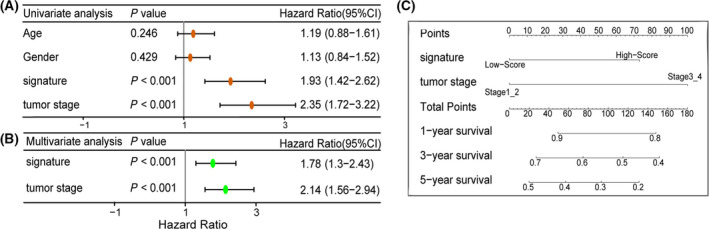
Independent prognostic value of prognostic signature. (A) Univariate Cox independent prognostic analysis. (B) Multivariate Cox independent prognostic analysis. (C) Nomogram to predict the 1‐, 3‐, and 5‐year OS of LUAD. Univariate and multivariate Cox regression analyses using the ‘survival’ R 4.0.3 package. Error bars, confidence interval (CI).

Univariate and multivariate Cox regression analyses were performed for risk signature to determine whether the signature had independent prognostic value. In univariate Cox regression analysis, prognostic signature and tumor stage were significantly associated with OS (*P* < 0.05; Fig. 4A). Multivariate Cox regression analysis showed that the prognostic signature could be used as an independent prognostic factor in predicting the OS of LUAD (*P* < 0.05; Fig. 4B). Prognostic signature and tumor stage are taken as the variables for nomogram, and the score of each variable is added to get the total score, which can be used to estimate the 1‐, 3‐, and 5‐year OS of LUAD patients (Fig. 4C).

### Different prognostic of 6‐signature gene

**Fig. 5:**
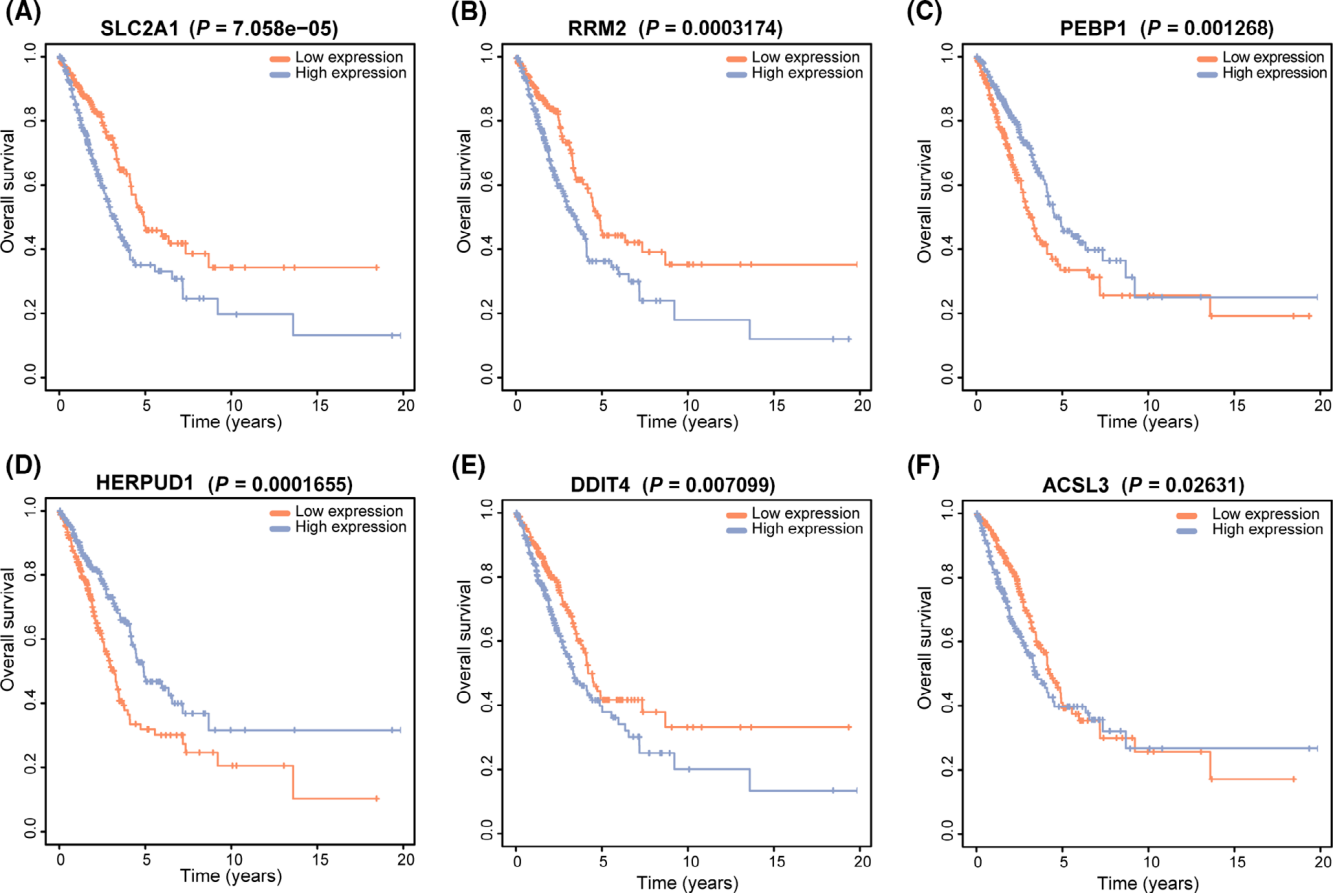
Survival analysis of six FRGs using two‐sided log‐rank test. (A) The OS analysis of SLC2A1 (*P* < 0.05). (B) The OS analysis of RRM2 (*P* < 0.05). (C) The OS analysis of PEBP1 (*P* < 0.05). (D) The OS analysis of HERPUD1 (*P* < 0.05). (E) The OS analysis of DDIT4 (*P* < 0.05). (F) The OS analysis of ACSL3 (*P* < 0.05).

The effects of the six signature genes on the survival of LUAD patients were analyzed, and patients were divided into high and low expression groups according to the median of gene expression values. The prognosis of SLC2A1 (*P* < 0.001; Fig. 5A), RRM2 (*P* < 0.001; Fig. 5B), DDIT4 (*P* = 0.007; Fig. 5E), and ACSL3 (*P* = 0.026; Fig. 5F) high expression group was poor, while that of PEBP1 (*P* = 0.0012; Fig. 5C) and HERPUD1 (*P* < 0.001; Fig. 5D) high expression group was better. This result was consistent with the expression heatmap of the six genes in Fig. 1, suggesting that patients with high expression of ACSL3, DDIT4, RRM2, and SLC2A1 had a high‐risk score and poor prognosis, while patients with high expression of PEBP1 and HERPUD1 had a low‐risk score and good prognosis.

### The expression patterns of 6‐signature gene at protein level

**Fig. 6:**
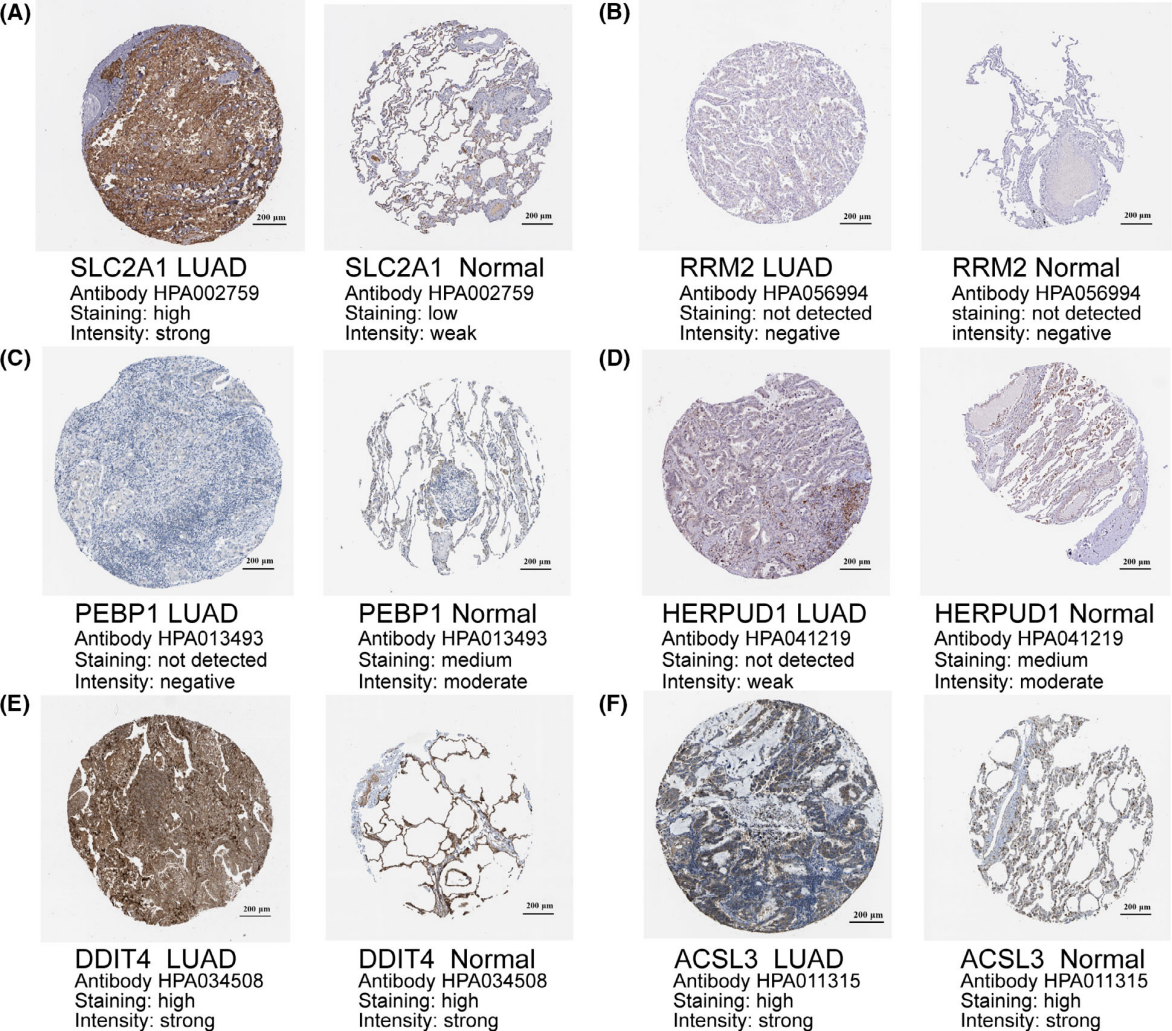
The protein expression levels of six FRGs in LUAD tumor tissues and normal tissues. (A) IHC staining of SLC2A1. (B) IHC staining of RRM2. (C) IHC staining of PEBP1. (D) IHC staining of HERPUD1. (E) IHC staining of DDIT4. (F) IHC staining of ACSL3. IHC, immunohistochemistry. Scale bars = 200 μm.

After detecting the prognosis of the six signature genes in LUAD, we further explored the protein expression patterns of the six signature genes in LUAD and normal tissues using the HPA database, and the results were shown in Fig. 5. IHC staining analysis indicated that ACSL3 (Fig. 6F) and DDIT4 (Fig. 6E) were highly stained in both LUAD and normal tissues, while RRM2 (Fig. 6B) protein was not expressed in either of them. SLC2A1 (Fig. 6A) was highly expressed in LUAD tissues and low in normal tissues. In addition, the protein levels of PEBP1 (Fig. 6C) and HERPUD1 (Fig. 6D) were not expressed in normal lung tissues, whereas medium expression levels of these genes in LUAD tissues.

### GO and KEGG pathway analysis

**Fig. 7:**
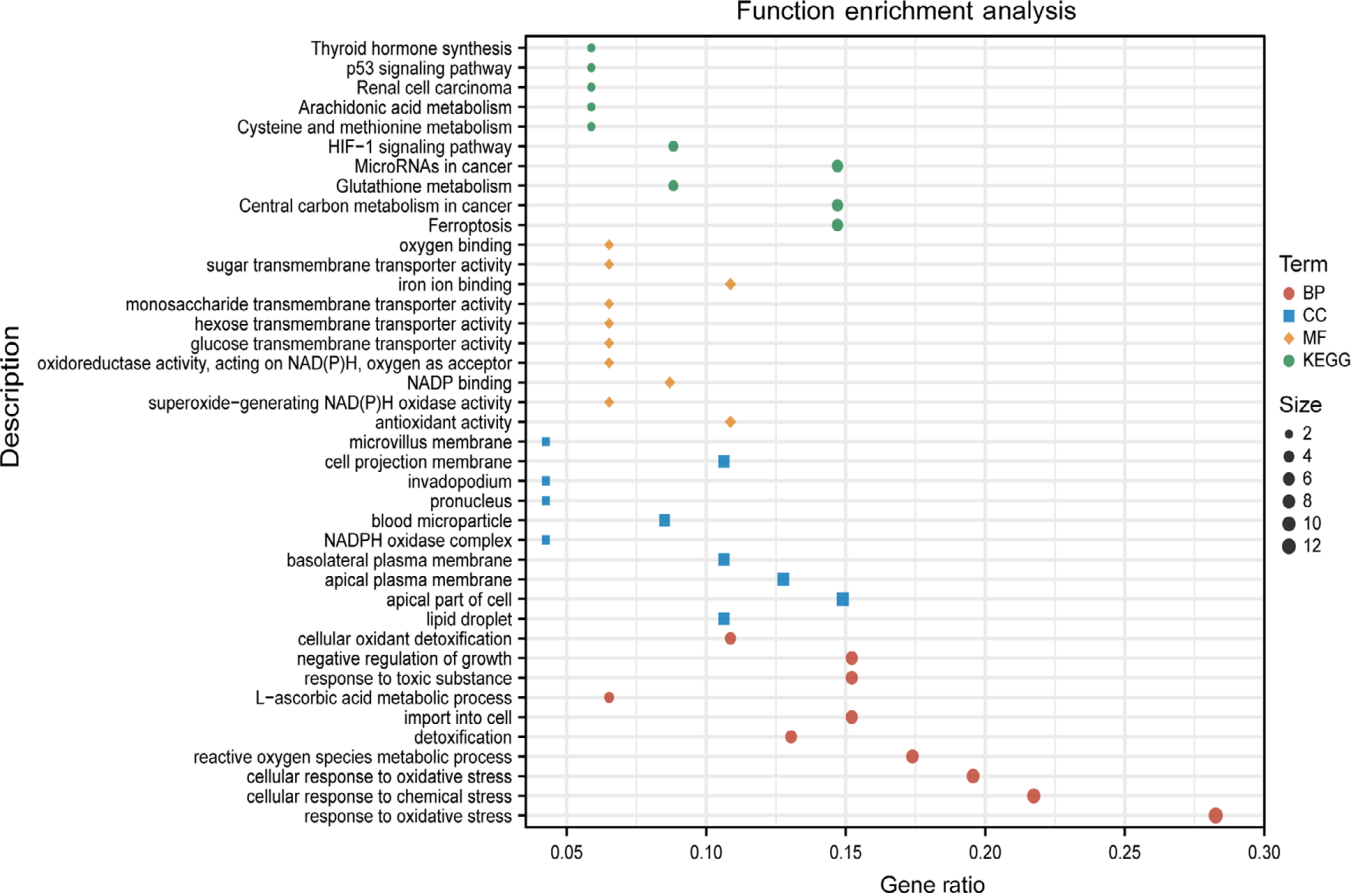
Functional enrichment analysis of FRGs. Top 10 terms of biological process. Top 10 items of a cellular component. Top 10 terms of molecular function. Top 10 terms of KEGG pathways. KEGG, Kyoto Encyclopedia of Genes and Genomes.

To investigate the potential biological functions and pathways between the high‐risk score group and the low‐risk score group, GO and KEGG pathway enrichment analysis were performed on differentially expressed genes (DEGs) between the two groups. Significantly annotated GO including biological processes (BP), cellular components (CC), molecular function (MF), and KEGG pathways of these DEGs are demonstrated in Fig. 7. GO enrichment showed that these genes were mainly enriched in ROS metabolic process, negative regulation of growth, NADPH oxidase complex, and iron ion binding, etc. KEGG pathway analyses indicated that these genes were mainly enriched in ferroptosis, HIF‐1 signaling pathway, arachidonic acid metabolism, cysteine and methionine metabolism, and p53 signaling pathway.

### Immune and clinical characteristic correlation of prognostic signature

**Fig. 8:**
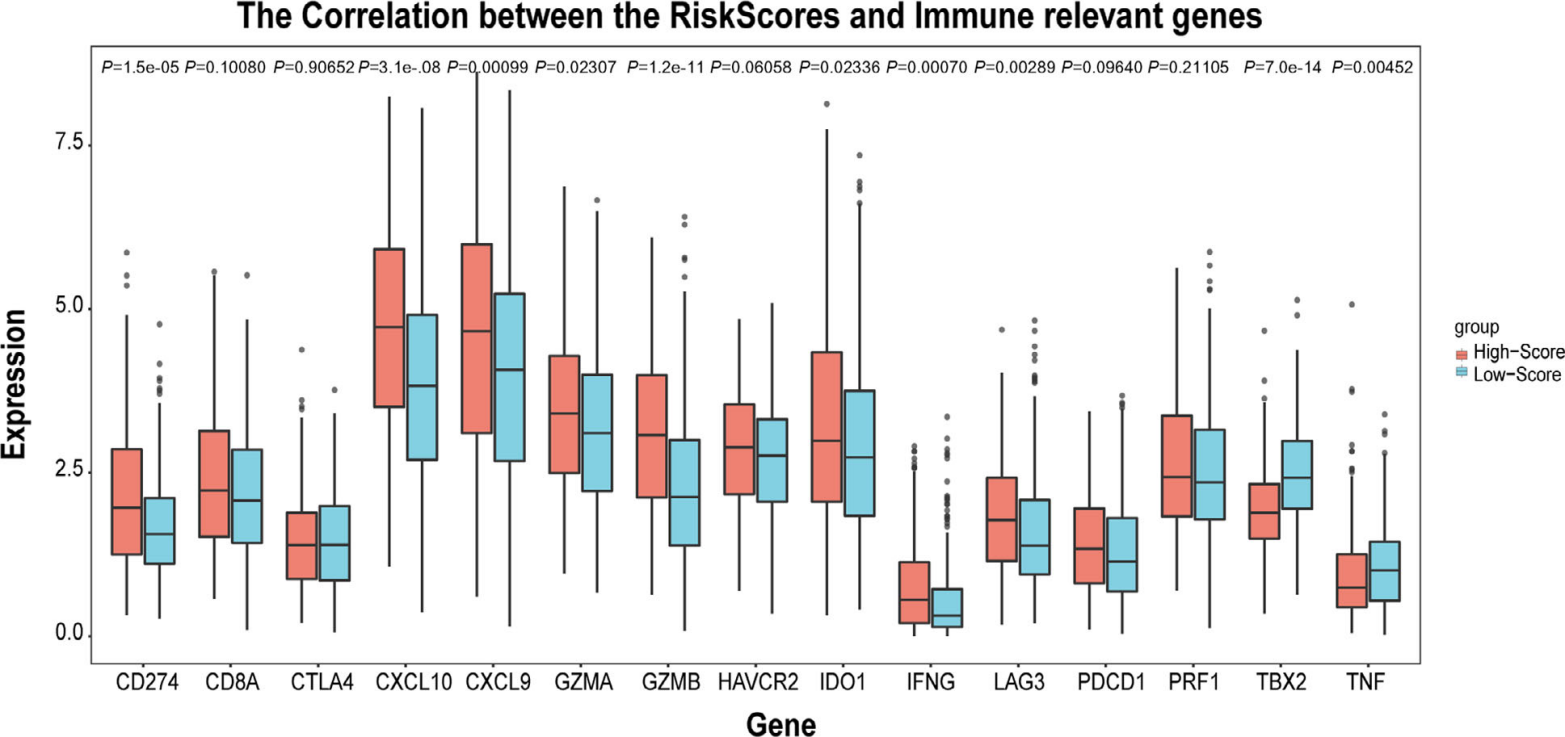
The relationship between risk score and immune‐checkpoint‐relevant signatures and immune‐activity‐related signatures. *P* values, Wilcoxon rank‐sum test.

**Fig. 9:**
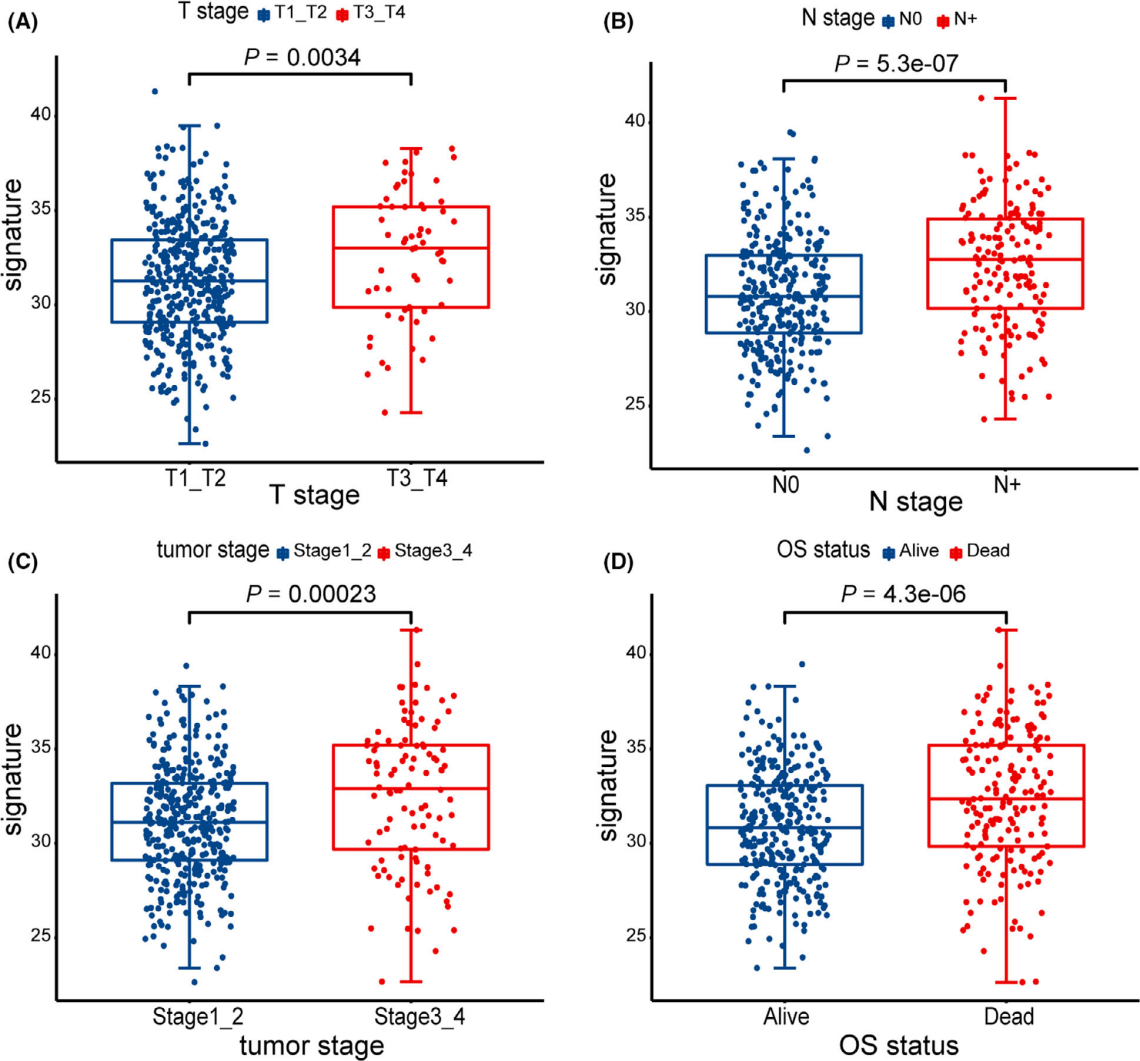
Clinical characteristic correlation analysis. (A) The clinical correlation between risk score and T stage (*P* = 0.0034). (B) The clinical correlation between risk score and N stage (*P* = 5.3e−07). (C) The clinical association between risk score and tumor stage (*P* = 0.00023). (D) The clinical correlation between risk score and survival status (*P* = 4.3e−06). *P* values, Wilcoxon rank‐sum test.

We verify the immune correlation and clinical characteristic correlation of prognostic signature. PRF1, TNF, GZMA, TBX2, CXCL9, CXCL10, GZMB, CD8A, and IFNG were selected as immune‐activity‐related signatures and PDCD1, HAVCR2, CD274, LAG3, CTLA4, and IDO1 as immune‐checkpoint‐relevant signatures. We observed that CD274, LAG3, GZMA, CXCL9, CXCL10, GZMB, IFNG, and IDO1 were significantly overexpressed in the high‐risk score group, while TBX2 and TNF were significantly overexpressed in the low‐risk score group, as showed by the Wilcoxon test (Fig. 8). Relationship between prognostic signature and clinical characteristic was subsequently analyzed. The results showed that the signature was significantly associated with T stage (*P* = 0.0034), N stage (*P* = 5.3e−07), tumor stage (*P* = 0.00023), and survival status (*P* = 4.3e−06). Patients with a high‐risk score were more likely to have large tumors size (Fig. 9A), lymph node metastasis (Fig. 9B), advanced tumor stage (Fig. 9C), and poor prognosis (Fig. 9D).

## Discussion

As a result of their growth, cancer cells require more iron than normal, noncancerous cells do. Iron dependence can make cancer cells susceptible to iron‐mediated necrosis, referred to as ferroptosis [13]. Ferroptosis differs from autophagy, apoptosis, and necrosis in terms of function and cell morphology, which mainly caused by the imbalance between the generation and degradation of lipid ROS in cells [31]. When the antioxidant capacity of cells is reduced, the accumulation of lipid ROS can cause oxidative cell death [5, 10]. Ferroptosis has attracted a lot of attention recently, especially since genes that initiate or execute necroptosis in cancers are downregulated and silenced [32]. A positive or negative regulation of ferroptosis can influence the treatment of ferroptosis‐associated disease: Induction or inhibition of ferroptosis may be effective for treating refractory tumors [33]. Emerging evidence shows that ferroptosis plays an important role in inhibiting tumorigenesis, especially against malignancies that are resistant to conventional therapies [7, 34, 35]. Increasing research on ferroptosis in various cancers suggests that ferroptosis is gradually being recognized as a potential form of cancer elimination [8, 36]. Several mechanisms that regulate ferroptosis are also being investigated. Yang *et al*. found that glutathione peroxidase‐4 (GPX4) is an inhibitor protein of lipid peroxidation process, which can degrade small molecular peroxides and some lipid peroxides, thus inhibiting lipid peroxidation. Therefore, inhibition of GPX4 will induce ferroptosis of cells [18, 37]. Inhibition of systemXc‐ can block the absorption of glutathione (GSH), resulting in reduced GPXs activity, decreased cell antioxidant capacity, accumulation of lipid ROS, lead to oxidative cell death and ferroptosis [5, 38]. By downregulating the expression of SLC7A11, p53 can inhibit the absorption of cystine by systemXc‐, resulting in the decreased activity of cysteine‐dependent glutathione peroxidase, decreased antioxidant capacity of cells, and increased lipid ROS, leading to ferroptosis of cells [39, 40]. Although the signaling pathways of ferroptosis occur in different ways, ultimately, ferroptosis occurs by directly or indirectly affecting the activity of GPXs and reducing the antioxidant capacity of cells, resulting in increased lipid peroxidation reaction and increased lipid ROS. Studies have shown that upregulation of GSH synthesis in NSCLC cells suppresses ferroptosis [41].

At present, multigene prognostic signature has been widely studied and applied in tumor prognosis analysis [23, 24]. In this study, we collected a series of FRGs to construct FRG‐related prognostic signature to explore and validate the potential value of FRG in LUAD. Although the prediction performance of the prognostic signature was not satisfactory in TCGA database (AUC_max_ < 0.7), it showed good prediction performance on GSE31210 and GSE72094 datasets. Therefore, we believe that the signature has good sensitivity and specificity for predicting the prognosis of LUAD and had independent prognostic value. In addition, the prognostic signature was significantly associated with several clinical characteristics (T stage, N stage, tumor stage, and survival status) as well as many immune‐activity‐related genes and immune‐checkpoint‐related genes, and the relationship between these FRGs and immunotherapy remains to be further studied. We further focused on the prognosis and protein level expression of six signature genes. Although high expression of ACSL3, DDIT4, and RRM2 was associated with a high‐risk score and poor prognosis, immunohistochemical staining suggests that there was no significant difference in ACSL3, DDIT4, and RRM2 staining between LUAD and normal tissue. Exogenous MUFAs require ACSL3 to prevent the accumulation of lipid ROS on the plasma membrane during ferroptosis. Low ACSL3 expression is associated with increased sensitivity to ferroptosis in all cancer cell types, exogenous MUFAs and ACSL3 activity specifically promote the cellular status of antiferroptosis [42]. Erastin can reduce the absorption of cystine by inhibiting systemXc‐, causing ferroptosis and significantly upregulating DDIT4, but further study needed to confirm the association between DDIT4 and ferroptosis [43]. RRM2 suppresses ferroptosis in liver cancer cells by promoting GSH synthesis and maintaining GPXS activity. Meanwhile, RRM2 could reverse erastin‐induced ferroptosis [44, 45]. High expression of SLC2A1 was associated with high‐risk score and poor prognosis, and immunohistochemical results also showed high expression in LUAD tissues and low expression in normal tissues. SLC2A1 mediates glucose uptake to promote glycolysis, pyruvate oxidation, and the tricyclic acid cycle, and to stimulate fatty acid synthesis and ultimately facilitate lipid peroxidation‐dependent ferroptosis [46, 47]. Patients with high expression of PEBP1 and HERPUD1 had a low‐risk score and good prognosis. Immunohistochemical staining showed that PEBP1 and HERPUD1 were not expressed in normal lung tissues but were medium expressed in LUAD tissues. The PEBP1/15LO complex leads to ferroptosis, in which the downregulation of PEBP1 was concerned with lowered sensitivity to ferroptosis [48, 49]. ZIP7 was essential for ferroptosis, and inhibition of ZIP7 protects ferroptosis. ER stress induced by erastin and associated with ferroptosis. ZIP7 depletion triggers ER stress and induces the expression of HERPUD1, which mediate an unknown process to protect ferroptosis. The knockdown of HERPUD1 eliminated the ZIP7‐inhibited ferroptosis protective [50, 51].

In conclusion, a novel prognostic signature consisting of six FRGs was successfully constructed, which has good sensitivity and specificity for predicting the prognosis of LUAD and had independent prognostic value, with further in‐depth research found that the prognostic signature was significantly associated with several clinical characteristics (T stage, N stage, tumor stage, and survival status) as well as many immune‐activity‐related genes and immune‐checkpoint‐related genes, indicating that their relationship with immunotherapy remains to be further investigation. Regrettably, there were some limitations in our study. Firstly, all the dada analyzed in our study were retrieved from the public databases that were not verified in specific clinical trials. Secondly, the role of these genes in LUAD prognosis needs to be further studied through *in vitro* and *in vivo* experiments.

## Conflict of interest

The authors declare no conflict of interest.

## Author contributions

Jing Zhou conceived and designed the study. Zhaona Li obtained the datasets. Jing Zhou and Xinyue Wang performed the bioinformatics analysis and wrote the manuscript. Richeng Jiang contributed to the experimental design and was responsible for revising the manuscript and approving the version to be published. All authors reviewed and approved the final manuscript.

## Data Availability

The datasets used and/or analyzed during the current study are available from the corresponding author upon reasonable request.
